# How do parents experience support after the death of their child?

**DOI:** 10.1186/s12887-016-0749-9

**Published:** 2016-12-07

**Authors:** Sandra Gijzen, Monique P. L’Hoir, Magda M. Boere-Boonekamp, Ariana Need

**Affiliations:** 1 Department HTSR, IGS Institute for Innovation and Governance Studies, University of Twente, PO Box 217, 7500 AE Enschede, Netherlands; 2 TNO Child Health, P.O. Box 2215, 2301 CE Leiden, Netherlands; 3 Department Public Administration, IGS Institute for Innovation and Governance Studies, University of Twente, PO Box 217, 7500 AE Enschede, Netherlands

**Keywords:** Bereavement care, Child mortality, Prevention

## Abstract

**Background:**

A child’s death is an enormous tragedy for both the parents and other family members. Support for the parents can be important in helping them to cope with the loss of their child. In the Netherlands little is known about parents’ experiences of the support they receive after the death of their child.

The purpose of this study is to determine what support parents in the Netherlands receive after the death of their child and whether the type of care they receive meets their needs.

**Method:**

Parents who lost a child during pregnancy, labour or after birth (up to the age of two) were eligible for participation. They were recruited from three parents’ associations. Sixty-four parents participated in four online focus group discussions. Data on background characteristics were gathered through an online questionnaire. SPSS was used to analyse the questionnaires and Atlas ti. was used for the focus group discussions.

**Results:**

Of the 64 participating parents, 97% mentioned the emotional support they received after the death of their child. This kind of support was generally provided by family, primary care professionals and their social network. Instrumental and informational support, which respectively 80% and 61% of the parents reported receiving, was mainly provided by secondary care professionals. Fifty-two per cent of the parents in this study reported having received insufficient emotional support. Shortcomings in instrumental and informational support were experienced by 25% and 19% of the parents respectively. Parental recommendations were directed at ongoing support and the provision of more information.

**Conclusion:**

To optimise the way Dutch professionals respond to a child’s death, support initiated by the professional should be provided repeatedly after the death of a child. Parents appreciated follow-up contacts with professionals at key moments in which they were asked whether they needed support and what kind of support they would like to receive.

**Electronic supplementary material:**

The online version of this article (doi:10.1186/s12887-016-0749-9) contains supplementary material, which is available to authorized users.

## Background

The death of a child is an enormous tragedy for both the parents and other family members. Parents experience intense feelings of loss after their child’s death [1]. The death of the child influences not only the family system, which is internally disrupted, [2, 3] but also others: neighbours, friends, relatives (i.e., the social network) and other acquaintances. Everyone needs to deal with his or her own grief. While parents try to pick up the pieces, support that meets their needs is important for them to cope with the loss of the child [3].

The period of mourning and the way people mourn differ from person to person. There is no “right” way of grieving [4, 5]. Some authors describe different stages in the grieving process, which may overlap each other [4]. Others state that grief is a complex process which has no stages and consider it to be more like a fingerprint: unique and erratic [6]. The dual process model, [7] in which an effective way of mourning is finding a balance between ‘loss orientation’ and ‘restoration orientation’, fits well with this view. Although people mourn in their own way, on different levels of intensity and time course, complicated forms of grief have been reported [8]. As many as 58% of parents who lost a child suddenly and unexpectedly, show 18 months after the death of their child, “complicated grief reactions” if the definition of Prigerson & Jacobs is used [9]. But given the nature of the parent-child relationship, this may not necessarily indicate pathological processes. Bereavement outcome depends on a complex interaction between situational, personal and coping factors [10]. It is known that grief rumination [11] leads to more symptoms of depression and complicated mourning [10]. Complicated grief then, like yearning existing longer than six months post-loss, [12, 13] increases the risk of psychosocial and psychiatric problems and death from natural and external causes [14–16]. To prevent psychosocial and psychiatric problems after the death of a child it is important that professionals understand the complex emotional grieving process and identify symptoms of possible complicated grief in parents and other family members at an early stage, in order to provide adequate family support.

The intensity of parental grief is related to a number of factors, such as gender and coping strategies of parents, the child’s age, and circumstances surrounding the death. Cultural and ethnic differences must be taken into account in assessing the extent of expressions of grief and mourning. What is considered normal in one culture may not be in another [17]. Mothers experience intensive grief reactions more often than fathers [14, 18–20]. Gender differences are also observed in the use of coping strategies in relation to death. It seems that women confront their emotions, while men use avoidance coping strategies more often. The intensity of grief among parents generally increases when the child dies at an older age [14]. Furthermore, parents experience more grief reactions when the death is due to an external cause and is unexpected. Features of grief and coping styles differ between individuals, different ethnic groups and cultural backgrounds [10]. This implies that the need for support also varies.

When their child has died, parents receive support from family, friends, colleagues and other people, for example from day care, school or sport clubs, and from (health) professionals. There are different types of support described in literature, [21, 22] which can be divided into emotional, instrumental and informational support. Emotional support is any behaviour in which empathy, love, trust and care is provided to parents. Instrumental support is the provision of tangible assistance or services that directly help parents. Informational support is the provision of advice and information, which empowers parents to make informed decisions about the care offered to their child, such as withdrawal of treatment, as well as other issues pertaining to family life [21]. Health professionals and others involved in a child’s death are confronted with their own emotions and fears. This may influence the way they approach the parents of a deceased child [23]. The care, or the lack of care, that parents receive around the time of death has a great impact on the adjustment process and well-being of the parents in the long-term [24]. In case of a sudden and unexpected death in particular, the initial care largely determines the course of bereavement. In this context professionals should realise that parents want to say goodbye to their child, receive information about the cause of death and feel supported by professionals [25]. Parents value health professionals and others who approach them with empathy, kindness and respect. They also value professionals when they listen and communicate well and offer support before and after the death of a child [15, 24–26]. According to parents, support should be offered on an individual basis and may vary in intensity depending on the family needs [15]. Support should not be focused solely on parents but also on any surviving siblings [23].

In the Netherlands, professionals from different organisations are involved when children die. In protocols, guidelines or other working agreements, supporting the family after a child’s death receives relatively little attention [27]. The Dutch Preventive Child Healthcare has a guideline particularly directed at counselling families after the death of a child [28]. For professionals in palliative care, a national guideline, ‘Grief’, is available and it describes how surviving relatives can be supported [29]. The Dutch Association of Pediatrics developed a guideline in collaboration with the Dutch College of General Practitioners specifically directed at the organisation of care for children in the palliative phase [30]. Other professionals have generic aspects of family support included in their guidelines.

Although there is a lot of knowledge on bereavement and increasing interest in the support of the family, information is lacking about parents’ experiences of the support they received after the death of their child. In this study we answer the research question: what bereavement care did parents in the Netherlands receive after the death of their child and did this care meet their needs? The answers to these questions can help professionals to optimise the support they offer after a child’s death.

## Methods

### Study design

Online focus groups and a questionnaire were used to explore what bereavement care parents in the Netherlands received after the death of their child. The METC Twente (Medical Ethical Committee Twente) reviewed the project plan for ethical permission, but decided the study was not subject to the Medical Research Involving Human Subjects Act (WMO) (METC/11011.boe) [31].

### Study sample

The target population consisted of parents who have lost their child during pregnancy and labour or after birth, up to the age of two. To recruit these parents we contacted the chairs of three parents’ associations by email: the Association of Parents of Cot Death Children (in Dutch: Vereniging Ouders van Wiegendoodkinderen), the Association of Parents of a Deceased Child (in Dutch: Vereniging van Ouders van een Overleden Kind) and the online Sweet Angel Foundation (in Dutch: Stichting Lieve Engeltjes). The Association of Parents of Cot Death Children is a support group that consists of fellow sufferers. Its aim is “to support parents and others who are closely involved, to give information, to gather knowledge on cot death and to stimulate research to optimally support families and to put research to prevent Sudden Infant Death Syndrome (SIDS) on the agenda” [32]. The Association of Parents of a Deceased Child is an organisation which consists of parents of a deceased child (of any age) that aims “to offer understanding and compassion to fellow sufferers” [33]. The Sweet Angel Foundation is an association for parents of a child that died during pregnancy, birth or at an older age, and other persons who are confronted with a child’s death in or outside the family. This association “provides fellow sufferers the opportunity to get in touch with each other by email” [34].

The chairs of the three parents’ associations agreed to invite their members to participate in the study by means of an invitation letter, which contained information about the objectives and procedure of the study. The 256 members of the Association of Parents of Cot Death Children received the invitation letter by post. The Association of Parents of a Deceased Child published the invitation letter in their newsletter, which is delivered to all members including the 200 who lost their child when he or she was under two. The Sweet Angel Foundation placed the invitation letter in their newsletter, which all members received by email. Respectively 33, 1 and 38 parents signed-up via e-mail.

### Data collection

Data were gathered through four asynchronous online focus group discussions in February and March 2013. Participating parents from the Association of Cot Death Children were divided into two focus groups of 16 and 17 persons. Participating parents from the Association of Parents of a Deceased Child and the Sweet Angel Foundation were divided into two focus groups of 20 and 19 persons.

Background characteristics of the participating parents were gathered by means of a questionnaire.

A semi-structured questionnaire was used to guide the focus group discussions. To conduct these online group discussions a secure forum licensed by TNO Child Health [35] was used. Each session was guided by two moderators (first and second authors). Parents gave their consent to participate at the beginning of the online focus group discussion, after they received a document by email that described the procedure of logging in on the secure forum. This document also contained communication rules. Anonymity for participants was ensured through the use of nicknames. The secure forum was accessible to the participants for one week. Each day, the first moderator posted a question on the forum, to which participants could respond at any time of day. Participants could also respond to each other if they wished. In total, seven questions were posted about the support parents had received in the period around and after the death of their child, and whether this care met their needs. Parents were asked to describe who was involved around the time of death of their child and whether they had received support from professionals or other people. If parents reported receiving support, they were asked to describe who supported them, what kind of support they had received and what their experiences were in relation to the support (see Additional file 1). The two moderators followed the discussion on a daily basis, in order to stimulate the exchange of information and experiences by answering participants’ questions when something was unclear. The second author (a psychotherapist) also referred some parents to a form of trauma therapy or a website for information when she felt this was appropriate.

### Data analysis

First, the background characteristics of the participants were categorised. Second, the input given in the online focus groups, saved on the secure forum, was analysed using Atlas ti [36]. A codebook was created based on the time period of support in relation to the death, the type of support (emotional, instrumental or informational) parents had received or lacked from a certain person, and wishes or recommendations from parents with regard to support. Support that was in line with the parents’ needs or expectations was identified as good practice when parents valued this explicitly with words. The first author coded all four online focus groups and the third and fourth author each independently coded two of the four online focus groups, to minimise the introduction of researcher selection bias into the results. Relevant text fragments related to the topics of the seven questions in this study were selected and given codes. The codes and the corresponding fragments coded by the different coders were compared. The differences were discussed between the three researchers. Ultimately, consensus was reached about the definitive set of codes and the fragments that corresponded to these codes. Next, the first author removed duplicates in codes and sorted the remaining codes by the kind of support that parents reported that they had received or lacked after their child’s death.

## Results

### Background characteristics of the participants

Of the 72 parents who had signed up for participation, 29 from the Association of Parents of Cot death Children, one from the Association of Parents of a Deceased child and 34 from the Sweet Angel Foundation actually participated in the online focus group discussions. Fifty-seven of these 64 participants completed the questionnaire on background characteristics (Table 1).

**Table 1:** Background characteristics of 64 parents^1^ participating in the online focus group discussions and of their deceased children

Characteristics	Participants N = 64	
	Number	%
Participating parent		
Mother	53	83
Father	4	6
Unknown	7	11
Ethnicity		
Dutch	57	89
Unknown	7	11
Church membership		
No	35	55
Yes	22	34
Unknown	7	11
Year of death of the child		
1970-1999	22	34
2000-2012	35	55
Unknown	7	11
Age of the child at time of death		
Stillbirth	10	16
First month	15	23
2nd -12th month	25	39
Second year	7	11
Unknown	7	11
Expected /unexpected death		
Expected	16	25
Unexpected	41	64
Unknown	7	11
Cause of death		
Pregnancy and childbirth related conditions	13	20
Congenital malformations, deformations and chromosomal abnormalities	10	16
Sudden infant death syndrome	26	41
Other	8	12
Unknown	7	11
Place of death		
Stillbirth	10	16
At home	24	38
In hospital	15	23
Other	8	12
Unknown	7	11

Seven parents who participated in the online focus group discussions did not fill out the questionnaire (answer category: ‘unknown’)

Most of the 64 participants were mothers (83%). Their mean age was 42.4 years, ranging from 24 to 65 years. All of them were Dutch. Their children died between 1970 and 2012; more than half of the children died after the year 2000. Sixteen per cent of the children died during pregnancy; 39% died between the ages of two months and twelve months. Sixty-four per cent of all deaths were unexpected. Forty-one per cent of the deaths were categorised as Sudden Infant Death Syndrome (SIDS); other causes of death were pregnancy and childbirth related conditions and congenital malformations, deformations and chromosomal abnormalities. Most children died at home (38%) or in the hospital (23%); eight children died elsewhere: three with family, friends or neighbours; four at the crèche, nursery or child-minder’s and one in a car seat.

### Parents’ experiences with support

The kind of support parents reported having received or lacked after their child’s death is shown in Table 2. An overview of the professionals who did or did not provide support, for each type of support, as reported by parents is given in Tables 3 and 4.

**Table 2:** Number of focus group participants who reported receiving or lacking support after the death of their child. The total number of participants in the focus groups was 64

Type of support	Number of participants who reported receiving support	Number of participants who reported lacking support
Emotional^a^	62	33
Instrumental^b^	51	16
Informational^c^	39	12
Unspecified	0	9

^a^Emotional support: any behaviour in which empathy, love, trust and care is provided to parents

^b^Instrumental support: provision of tangible assistance or services that directly help parents

^c^Informational support: provision of advice and information, which empowers parents to make informed decisions about the care offered to their child as well as other issues pertaining to wider family life

**Table 3:** Specification of the persons/organisations who/that gave support to the parents after the death of their child, as reported by the 64 focus group participants

Person/organisation who/that gave support	Number of participants who reported receiving support after the death of their child		
	Emotional	Instrumental	Informational
Health care professionals			
Preventive health care	7	5	1
Primary care^a^	43	23	9
Secondary care^b^	33	35	29
Maternity care outside the hospital	15	9	5
Acute care outside the hospital	5	2	2
Mental health care	28	4	3
Other professionals			
Funeral service	10	20	12
(Pre)school-related care	3	0	1
Work-related care	8	8	0
Informal network			
Partner	17	1	0
Family	49	22	5
Social network	38	18	2
Support groups	18	6	7
Other^c^	7	10	6

^a^Primary care: general practitioner, social worker and home care nurse

^b^Secondary care: paediatrician, gynaecologist, other medical specialist, nurse, personnel of the Accident and Emergency department

^c^Other: media, photographer and people not specified by parents

**Table 4:** Specification of the people/organisations who/that did not give support to the parents after the death of their child, as reported by the focus group participants

Person/organisation who/that did not give support as perceived by the respondents	Number of participants who reported lack of support after the death of their child		
	Emotional	Instrumental	Informational
Health professionals			
Preventive health care	2	1	0
Primary care^a^	5	1	0
Secondary care^b^	6	2	5
Maternity care outside the Hospital	1	2	1
Acute care outside the hospital	0	0	0
Mental health care	5	0	0
Other professionals			
Funeral service	1	1	1
(Pre)school-related care	1	0	0
Work-related care	3	1	0
Informal network			
Partner	0	0	0
Family	8	0	0
Social network	4	0	0
Support groups	0	0	1
Other^c^	11	9	7

^a^Primary care: general practitioner, social worker and home care nurse

^b^Secondary care: paediatrician, gynaecologist, other medical specialist, nurse, personnel of the Accident and Emergency department

^c^Other: media, photographer and persons not specified by parents

#### Emotional support

Of the 64 parents, 62 (97%) mentioned the emotional support they received after their child’s death (Table 2). Emotional support was mainly provided by family, primary care professionals (i.e., general practitioner, social worker and home care professional) and the parents’ social network (Table 3). Examples of good practices are illustrated in the following quotes:

“*We were very satisfied with the support of the general practitioner who did everything for us to sort out everything around the death of our child.”* (Year of death, 1997)

“*The general practitioner often visited us or called us sometimes to see how we coped. We knew that we could always contact her for questions and that thought was comforting*.” (Year of death, 2010)

*“Our parents and the rest of the family were there for us to provide a shoulder to cry on, to listen to us and ask how we were coping. This kind of support is priceless and has been very crucial for us.”* (Year of death, 2010)

Despite the fact that most parents received emotional support, 33 out of the 64 parents (52%) reported lacking this kind of support (Table 2). Parents reported a lack of emotional support in particular from other (not specified) persons and family (Table 4). The following quotes illustrate the kind of emotional support two parents had missed:

“*Like my mother-in-law subtly noted after 6 weeks “Are you still crying? You have to stop doing that now, because for us it is very annoying”. And yet she was a very sweet woman who did not know better*.” (Year of death, 1985)

*“Although we received a lot of support from our family, they do not know how it feels when you have lost a child. They completely miss the point in giving well-intentioned advice.”* (Year of death, 1997)

#### Instrumental support

Fifty-one of the 64 parents (80%) mentioned the instrumental support they received after their child’s death (Table 2). Instrumental support was particularly provided by primary and secondary care professionals (paediatrician, gynaecologist, other medical specialist, nurse, personnel of the Accident and Emergency department) and family (Table 3). Examples of instrumental support are reflected in the following quotes:

*“We received a lot of support from our family, who took over our household and made dinner for us. I have experienced this as pleasant.”* (Year of death, 1999)

“*The forensic physician allowed us to bring our daughter to the hospital ourselves without police or hearse. The hospital was informed about our arrival. A special room was prepared for us where we could stay. They offered us the opportunity to be present during the first examination, which we did not want to. After the examination we could take our daughter in our arms until she was taken away for the complete autopsy. Afterwards we put her in her own bed underneath a blanket as if she was going to sleep. We experienced this as a very warm gesture to our daughter and ourselves*.” (Year of death, 2005)

“*The hospital had organised a memorial service 5 months after the death of our daughter for all the parents of children that died at the neonatology department that year. The memorial service was followed by a get together with fellow sufferers. I am positive about this kind of support (as far as you could speak in those terms)*.” (Year of death, 2005)

Sixteen of the 64 parents (25%) mentioned a lack of instrumental support after the death of their child (Table 2). Parents reported a lack of instrumental support in particular from other (not specified) persons (Table 4). The following quote illustrates the kind of instrumental support one parent reported lacking:

*“After the death of our child we have had to struggle to get the help we needed. A psychologist with experience in bereavement was hard to find.”* (Year of death, 2011)

#### Informational support

Of the 64 parents, 39 (61%) mentioned the informational support they received after the death of their child (Table 2). Informational support was particularly provided by secondary care professionals (Table 3). The following quotes illustrate the informational support received from secondary care professionals:

*“We experienced the counseling for a future pregnancy in the hospital as very valuable. You are no longer the ‘unconcerned’ parent.”* (Year of death, 1993)

“*Both hospitals where I stayed were very supportive, especially one physician: the gynaecologist. The talks, the time, the personal advice. It was all well meant and direct. Although I did not want to hear it, he gave advice anyway. But I appreciated (and I still do appreciate) the support, the honesty and sincerity of this man*.” (Year of death, 2012)

Twelve out of 64 (19%) mentioned a lack of informational support after their child’s death (Table 2). Parents reported a lack of informational support in particular from other (not specified) persons and secondary care professionals (Table 4). The informational support that parents lacked is reflected in the following quotes:

*“At a follow up check the gynecologist told me that I should be pregnant again as soon as possible. This would not happen the next time. I did not get any further information.”* (Year of death, 1970)

*“For advice and information you have to look on the Internet.”* (Year of death, 2012)

### Recommendations of parents

Twenty of the 64 parents (31%) responded to the question about the ways in which support could be improved and what kind of support they had appreciated from which person. The recommendations they provided are directed at emotional, instrumental and informational support after the death of a child, as presented in Table 5.

**Table 5:** Recommendations reported by parents per type of support

Type of support	Recommendations
Emotional	Create possibility to share grief and experiences and get support not only after the death of a child but in the next pregnancy as well [1] [*year of death 1986*] [realized by the Care of Next Infant program (CONI)]
	Close relatives or friends should let the parents know that support could be provided anytime [1] [*year of death 2005*]
	Professionals should realise that parents want to hold and cuddle their deceased child [1] [*year of death 2005*]
	A physician (e.g., the GP), midwife or social worker should offer a consultation 6-12 months after the death of a child to check whether there are questions or whether parents need support [3] [*year of death 2005, 2011, 2012*]
	The GP or Preventive Child Health nurse should contact (phone, home visit) parents as a ‘safety net’ [1] [*year of death 2012*] several times after the death of their child to pay attention to the loss, listen to them [4] [*year of death 2000, 2010*] and signal problems in the grieving process at a very early stage [1] [*year of death 2010*]
	A hospital professional, like the gynaecologist or nurse, should contact parents uninvited to evaluate [2] [*year of death 2008, 2012*]
	Professionals should take into account the mental situation of the mother when she gives birth to a deceased child [1] [*year of death 2012*]
Instrumental	The GP should offer support and discuss his/her options for giving after care shortly after the death of a child [3] [*year of death 1985, 1997, 2000*]
	Professionals should structurally draw the parents’ attention to contact with fellow sufferers [2] [*year of death 2005*][still does not happen always]
	Support should be offered repeatedly by a professional from the hospital, midwife, preventive child health care professional or GP, especially when support from social network has stopped [2] [*year of death 2005, 2012*]
	Hospitals should organise a memorial service for all deceased children [1] [*year of death 2008*][happens in many hospitals, nowadays]
	Offer a form of maternity care once a week for 6 to 12 months [1] or help in the household for 1 year after the death of a child, to be reimbursed by the insurance company[1] [*year of death 2011*]
Informational	Professionals should draw parents’ attention to books, websites, documents [3] [*year of death 1997, 2005*]. A brochure that contains different kinds of support with contact information of professionals should be offered as a standard procedure shortly after the death of a child [1] [*year of death 2010*]
	The undertaker should provide parents with information about options for a funeral or cremation, including examples of grave covers and sample texts for cards [2] [*year of death 1997, 2005*][Is realized nowadays]
Unspecified	Lay down rules for bereavement leave for the duration that is needed [1] [*year of death 2011*]
	The hospital should offer a return visit to the department of the hospital where the child is born to speak the nursing staff [1] [*year of death 2012*]

## Discussion

When a child has died, many people are involved and provide some form of support to parents. Through the use of online focus group discussions we explored parents’ experiences with support after the death of their child aged two or younger.

Most parents mentioned the emotional support they received after the death of their child. This kind of support was particularly provided by family, primary care professionals and the parents’ social network. Instrumental and informational support was mainly provided by secondary care professionals. As described in other research, physicians arrange follow-up meetings, usually after 6 weeks, with parents to inform them about the autopsy findings, cause of death and genetic risk, to answer questions and to offer and provide support in the following pregnancy if needed [37].

An important finding is that slightly more than half of the parents reported a lack of emotional support, particularly from family. Furthermore, informational support from secondary care professionals was evaluated as insufficient and many parents experienced shortcomings in the instrumental and informational support of other, non-professionals.

Bereavement care has changed over time. In the post-war years parents were not allowed to talk about their deceased child, to see their child after death or to show their grief [38, 39]. Nowadays, there is a greater understanding of the loss and pain parents experience after the death of their child. Although this has changed the way in which support is provided to the family, parents in this study have made some recommendations to optimise family support. Parents emphasise that they would like to be approached with empathy and be acknowledged in their bereavement. Alongside this, health care workers should offer support repeatedly and provide parents with information about the grieving process and options for support. Parents appreciate contact with professionals six to twelve months after their child’s death, to check whether the family needs any extra care or support. This contact should be initiated by the professional. In line with the results of other studies, parents indicate that they would appreciate the provision of more support and follow-up appointments or contacts with a professional after the death of their child [25, 26].

### Strengths and weaknesses of this study

For our target population, the use of online group forums proved to be a comfortable form of group discussion. This may have helped with recruitment, because participants were confident that anonymity was guaranteed and they could decide when and where they wanted to answer the questions. We were able to recruit 64 respondents living throughout the country, of whom 57 provided information about the time, place and cause of death, the extent to which the death was expected, and the age of the child. However, parents were only recruited from support groups, which creates bias. It could be that parents who are members of support groups experience less support from family or have less or more coping skills than bereaved parents who do not participate in such a group. Recruitment through an invitation letter in the organisation’s newsletter seemed to be less effective than a letter sent by post. The low participation rate for parents from the Association of Parents of a Deceased Child might relate to the fact that this association includes parents of children who died at any age, while this study focusses only on young children. Furthermore, in the interpretation of the number of members of the parents’ associations it should be taken into account that membership lists usually include many dormant members. The distribution of the background characteristics of participants (mostly mothers of Dutch ethnicity) limits the generalisability of the results to athers or other ethnicities. In addition, we also were not able to observe gender differences in grief reactions and the way professionals should respond to this. With regard to church membership, the numbers are not remarkably different from the current Dutch population [40, 41].

The number of participants prohibits analysing subgroups according to the circumstances of the child’s death or parents’ characteristics. In addition to the small number of participants, the heterogeneity of time and circumstances of loss as well as the range of professionals likely to be involved in providing support, make it difficult to assess the internal validity of conclusions drawn from parents’ reports. The findings of this study shed light on Dutch practice over decades and do not provide a clear picture of current practice. Although participants provided valuable recommendations with regard to the way in which support should be improved, some of these have already been implemented in practice. We therefore recommend repeating this study with a larger sample size covering a short time span, for example the past five years, arranged by age of the deceased child and manner of death.

An advantage of online focus groups is that data do not need to be transcribed. This improves the accuracy of data and eliminates transcript bias, thereby increasing the quality of data [42]. A limitation of the online method is the varying response rate and length of responses to each individual question posted on the forum. Not every participant answered every question and was specific enough, which is understandable because it calls for a high degree of discipline. If we had been able to ask each parent to respond to each question posted on the forum, this would probably have resulted in a higher response rate and a more complete overview of the support parents received or lacked after the death of their child.

## Conclusion and recommendations

Different types of support are provided to parents after the death of their child. Although increasing attention has been paid to supporting families after the loss of a child, one-fifth to slightly more than half of the parents in this study lacked some sort of support or experienced support that was not in line with their needs or wishes. According to the results of this study, support initiated by professional should always include listening to parents and asking them at key moments after their child’s death whether they need (extra) support and what kind of support they would like to receive. Parents should also be asked specifically about the emotional support they receive from their family and their social network. When they lack this type of support, caregivers should explore with them how to reach out and receive more support. Furthermore, adequate communication skills and a respectful attitude are necessary in approaching the parents of a deceased child. The results of this study may not apply to every parent who has lost a child, because participants were a selected, self-admitted group. Future study is necessary in which parents are contacted through hospitals or government registries of death in order to compare the responses of those who participate in support groups and those that do not. Next to this, further research with the use of online focus groups is desirable, because the scope to reach parents and to include them in research seems so much wider than traditional focus groups.

## Additional file

Additional file 1: Seven questions that are posted in the online focus groups. Seven questions about the support parents received is written out. (DOCX 15 kb)

## Abbreviations

METC: Medical ethical committee
SIDS: Sudden infant death syndrome
SPSS: Statistical package for the social sciences
TNO: Applied scientific research
