# Supplementary material for: How do parents experience support after the death of their child?
Source: BMC Pediatr. 2016 Dec 7;16:204. doi: 10.1186/s12887-016-0749-9 (PMC5142355; doi:10.1186/s12887-016-0749-9)
Supplement: Additional file 1: — Seven questions that are posted in the online focus groups. Seven questions about the support parents received is written out. (DOCX 15 kb) [file 12887_2016_749_MOESM1_ESM.docx]

| Question 1. Who were involved in the care before and after the death of your child? |
| --- |
| Question 2. Who offered you support in the period around and shortly after the death of your child? |
| Question 3. Could you specify what kind of support (emotional, instrumental, informational) you received after the death of your child and from whom? |
| Question 4. Did professionals take into account the specific situation of your child or your cultural background? Did you know if professionals tuned in the support with each other? |
| Question 5. Were members of the direct or extended family involved in the support? |
| Question 6. At what moment started professional support and how long did it take? What kind of support did you receive and how often took this kind of support place? |
| Question 7. If you look back, what went well or could have been better with regard to the support you have received? What did you appreciate the most about the support and from whom? |

**Additional file 1. Seven questions that are posted in the online focus groups**
